# The effectiveness of peer support from a person with lived experience of mental health challenges for young people with anxiety and depression: a systematic review

**DOI:** 10.1186/s12888-023-04578-2

**Published:** 2023-03-24

**Authors:** Magenta B. Simmons, Sharla Cartner, Roxxanne MacDonald, Sarah Whitson, Alan Bailey, Ellie Brown

**Affiliations:** 1 Orygen, Parkville, VIC Australia; 2 Centre for Youth Mental Health, The University of Melbourne, Parkville, VIC Australia

**Keywords:** Peer support, Peer worker, Mental health, Early intervention, Youth, Anxiety, Depression

## Abstract

**Background:**

Peer workers support individuals experiencing mental health challenges by drawing on their shared lived experience. Peer support has become increasingly popular for young people with anxiety and depression, but the evidence base is unclear. This systematic review aimed to understand the effectiveness of peer support for youth depression and anxiety (either primary or comorbid), and to understand in which contexts, for whom, and why peer support works.

**Methods:**

A systematic search was conducted with the Orygen Evidence Finder, Embase, MEDLINE, and PsycInfo from January 1980 to July 2022. Controlled trials of interventions to improve mental health in young people (mean age 14–24), delivered by a peer worker with lived experienced of mental health challenges were included. Outcomes related to depression or anxiety were extracted and descriptive synthesis was undertaken due to the heterogeneity of studies. Study quality was rated using the Critical Appraisal Skills Programme; reporting adheres to the Preferred Reporting Items for Systematic Reviews and Meta-Analyses (PRISMA) statement.

**Results:**

Nine randomised controlled trials with 2,003 participants were included, with seven undertaken in high income countries. One targeted depression and anxiety, two stigma-distress (any mental disorder), one first episode psychosis, four studies preventing eating disorders and one drug misuse. One study successfully reduced anxiety and depression, another reduced depression only, four reported reductions in negative affect, with the final three measuring, but not having a significant impact on depression. Study quality was rated as ‘good’ overall.

**Discussion:**

Despite the uptake of youth peer support globally, there is limited evidence from controlled trials of the effect of peer support-related interventions on anxiety and depression. There is some effect on negative affect, especially for university students. Further rigorously designed trials of peer delivered interventions for young people need to be conducted with a focus on understanding the mechanisms of action underpinning peer support.

**Supplementary Information:**

The online version contains supplementary material available at 10.1186/s12888-023-04578-2.

## Background

Depression and anxiety are prevalent mental health disorders, with onset commonly occurring during adolescence and early adulthood [1]. In an international meta-analysis, 73.3% of people with an anxiety or fear-related disorder, and 34.5% of people with a mood disorder, had an onset by 25 years of age [2]. According to the World Health Organisation [3], the point prevalence of anxiety disorders was 3.6% in young people aged 10 to 14 years and 4.6% in those aged between 15 and 19 years, while the point prevalence of depression disorders was 1.1% and 2.8% in young people aged 10 to 14 years and 15 to 19 years, respectively. Twelve-month prevalence continues to increase with age; for example, in an international survey of 14,000 university students, around 18.5% experienced major depressive disorder and 16.7% generalised anxiety disorder [4]. The COVID-19 pandemic has likely contributed to increased distress and mental health symptoms. International data collected during the pandemic from a general youth population (≤ 18 years) demonstrated the prevalence of depression and anxiety symptoms that exceeded clinical cut-offs was 25.5% and 20.5% respectively [5]. However, this study reported symptoms rather than confirmed or probable diagnoses. In young adults (18 to 34 years) during the pandemic, the point prevalence of probable anxiety and depression diagnoses were 31.5% and 29.6%. While some young people receive help from trained professionals, a large proportion experience barriers to accessing services or do not have their needs fully met by services [6]. For those who do seek help, peers (i.e. similar-aged individuals in the young person’s social group without formal training) play a critical part in the help-seeking process for young people, who often turn to friends and family first before accessing formal help [7].

The degree to which informal support from peers, such as friends and acquaintances, is helpful will depend on how capable and willing peers are to provide such support. Attempts have been made to formalise peer support for promoting mental health and wellbeing in educational settings [8]. Although peer support can occur across multiple settings such as schools and specialised services (e.g., homelessness support), there is a rapidly growing peer workforce in mental health services, especially in high income countries [9]. Peer workers (also called peer support workers, peer practitioners and other terms) often work within mental health services, and are trained to draw on their lived experience of mental health challenges to deliver peer support. Peer workers are distinct from informal peers, who have not received training, are not employed to deliver peer support, and typically provide informal support to an *existing* personal connection (e.g. a friend or family member). Yet, peer workers are also distinct from traditionally qualified experts. In contrast to other roles in the mental health system, the peer worker and peer relationship is uniquely characterised by shared experience; the value of expertise through experience rather than clinical education and training; and reciprocity/ mutuality, whereby both individuals have the opportunity to intentionally learn and benefit from the relationship [10]. Peer workers are responsible for establishing and continually negotiating the ‘rules’ and power structures of the relationship, unlike a clinician-patient relationship [11]. Five common mechanisms have been identified across various models of peer support: *lived experience*; *love labour*, which refers to assurance of the emotional safety and wellbeing of peers; *liminality* of the peer worker, describing their position between identities of ‘patient’ and ‘clinician’; *strengths-focussed social and practical support*; and the *helper role* of the peer worker, which can facilitate their own recovery [12].

However, mental health services are not always favourable settings for peer workers. A number of barriers to implementation have been identified, including role confusion (i.e. employers and/or peer workers not knowing what the role is and how it fits within the service), role diffusion (i.e. spending time doing non-peer support tasks), co-optation (i.e. tasks becoming clinical in nature), professional stigma (i.e. negative attitudes from others and lack of credibility), and lack of support (i.e. availability of peer supervision, appropriate training and professional development) [13, 14].

Despite challenges, several reviews and meta-analyses have assessed the effectiveness of peer support interventions for adults with mental health challenges, finding that client and program characteristics varied widely [15–18]. For example, peer workers delivered a range of services, such as peer education, peer support, mentoring, psychoeducation, and case management, in different settings and mediums (see Table 1 for examples of peer support). Regardless, peer support interventions appear to be effective at improving hope, empowerment, increasing patient activation and self-efficacy [15, 16]. While one review [18] did not report a significant difference in hope, they suggest this could be attributed to the limited number of included studies that focused on this, and differences in methodologies and outcome measures.

**Table 1:** Different Real-World Models of Peer Support

Model	Description	Delivery Methods	Specific Model Benefits	Example
One-on-one (individual)	Peer Support between two people. Most likely involving a professional third-party to link the two people together	Face to face, Phone, Online	Tailored for the individual	One-on-one Peer Support Appointments (Orygen; https://oyh.org.au/client-hub/peer-support-team/1-1-peer-support-appointments)
Group peer-to-peer support	Groups share a lived experience. May be structured and organised, however, no formal facilitator. Can be independent or tied to a larger network	Face to face, Online	Tailored to the shared lived experience. Can be informal and include social activities	Grow (https://www.grow.org.au/)
Peer-led groups	Peer-Leaders sharing their lived experience to support and educate others similar to themselves. Can be workshops or structured group peer support often tied to a larger network	Face to face, Online	Tailored to the shared lived experience, may have educational aspects	Hearing Voices Network (https://www.hearing-voices.org/)
Groups co-facilitated by peer and traditionally qualified expert (e.g. clinician)	Often involves professional health services, where a group of people with a shared lived experience are supported by both an expert and peer. Can be structured and formal	Face to face, Online	Tailored to both group and individual, may have educational and treatment aspects	Headspace centres (https://headspace.org.au/headspace-centres/sunshine/youth-peer-support-at-headspace-sunshine/)
Online peer support	Can be one-on-one or group format. May have professional involvement through moderators. Mostly peer-to-peer support through forums	Online	Can be anonymous	Side by Side (Mind; https://sidebyside.mind.org.uk/)

Adapted from: https://www.nationalvoices.org.uk/peer-support-hub/peer-support-models-explained

NB: All models may be provided in either a traditional service (e.g., community mental health service) or peer-led service; additionally, all models may be designed for mental health challenges in general or specific concerns (e.g., vocational peer support).

Peer support interventions in adult populations generally did not impact quality of life, overall symptom severity, social inclusion [15], depression and anxiety symptoms [16], measures of hospitalisation [16], or service satisfaction [18]. Mixed results were reported for several outcomes, including service use [15–18] and client ratings of the working relationship [17, 18]. However, more recent evidence [19] has demonstrated a reduction in 12-month rate of readmission to acute care following a self-management program delivered by peer workers after patients had left the care of mental health crisis teams. The intervention also increased time until first readmission. This recent evidence suggests that peer support may reduce hospitalisations, an important objective outcome for health services worldwide. Trachtenberg (2013) [20] found that peer support significantly reduces hospital bed use, with the average financial savings outweighing additional costs of employing peer workers (benefit:cost ratio of 4.76:1), highlighting the cost-effectiveness of peer support.

While these reviews focused on services for individuals with ‘severe mental illnesses’ (typically psychoses, bipolar disorder, severe depression), several meta-analyses have assessed peer support specifically for ‘common’ mental health disorders (e.g. depression and anxiety). Pfeiffer, Heisler [21] included studies comparing peer support versus treatment as usual (TAU) or group cognitive behavioural therapy (CBT) for adults experiencing depression. The peer support group demonstrated a greater reduction in depression scores compared to TAU, but not significantly different to CBT, suggesting possible efficacy at the level of established treatments [21]. However, there was wide variability in patient populations, with many studies focusing on subpopulations, such as perinatal mothers. Similarly, Huang, Yan [22] reviewed randomised controlled trials (RCTs) of women with perinatal depression who received either peer support or TAU. For those who received peer support, depression scores were lower than controls, most participants reported intervention satisfaction, and it was cost-effective. Likewise, Field, Diego [23] reported that in two groups of randomly-assigned women with prenatal depression, one which received group peer support and the other received group interpersonal psychotherapy, both groups of women demonstrated significantly lower depression symptoms and cortisol levels (with a greater decrease in cortisol for the peer support group), despite the former group having a lower socio-economic status (SES), higher baseline depression scores, and shorter group sessions. Altogether, the available evidence suggests that peer work is a safe, effective, flexible and cost-effective intervention for adults, which promotes hope, empowerment, patient activation and self-efficacy, and reduces hospitalisations.

Previous work has focused foremost on adult populations, and there is a paucity of literature regarding young people. Using both peer-reviewed and grey literature, Gopalan and colleagues (2017) [24] undertook a United States-specific scoping review of youth peer support services and research for young people under 25 years old with emotional or behavioural problems. In total, 43 articles were identified, which included only three randomised controlled trials. The studies employed different peer support models, had different program goals, varying degrees to which peer workers were involved, and varying duties that peer workers undertook. There was also variation within the peer worker roles, including core competencies, training and supervision received by peer workers. Outside of the USA, the CHOICE project [25, 26] in Australia found that in a youth mental health service where the majority of clients present with anxiety and/or depression, participants (aged 16 to 25 years) who received the intervention (use of a co-designed shared decision-making tool with support from a youth peer worker) reported feeling more involved in treatment decisions compared to the comparison group. While the study did not measure anxiety and depression symptoms, the findings support the beneficial application of youth peer support in promoting shared decision-making.

While the majority of work in this area has focused on providing peer work within existing mental health services, an additional challenge is that many young people do not access formal clinical services for their mental health concerns [27]. Given that connections with peers are especially important during this developmental period, formal and informal peer support may represent an alternative avenue for young people to access support. For example, Reavley et al. (2011) [7] surveyed 275 young people with a mental health disorder in Australia to examine factors related to help-seeking and self-help behaviours. Participants most frequently sought help from family (77% of respondents) and close friends (73%), more so than general practitioners (53%). Peer workers may act as a bridge between, for example, untrained friends and accessing mental health services.

Therefore, peer support is a strong candidate intervention for young people with depression and anxiety, as people in this age group experience high rates of such concerns, and they are likely to be more receptive to seeking help from peers before or during engagement with formal clinical services. The primary aim of this systematic review was to understand the effectiveness of peer support for youth depression and anxiety (aged 14–24) as either a primary or comorbid mental health complaint. We also aimed to examine: 1) the contexts (e.g., geographical location, setting, format) in which peer support works; 2) who it does and does not work for; and 3) how it works.

## Methods

### Search strategy and information sources

This systematic review involved two search strategies. First, we utilised the Orygen Evidence Finder (OEF) database [28]. The OEF is a repository for all available randomised and non-randomised controlled trials, systematic reviews and meta-analyses that evaluate prevention and treatment strategies for common mental disorders and related challenges that have their peak onset during adolescence and early adulthood (mean age 12–25). These include depression, anxiety, bipolar disorder, substance use disorder, eating disorders, psychotic disorders, and self-harming behaviours. Its purpose is to provide a comprehensive evidence map of available intervention trials and reviews, highlighting where research gaps exist, and supporting knowledge translation (e.g., bibliographic database for mapping, scoping, and systematic reviews). The OEF is populated using systematic search and screening methods [28, 29]. Briefly, reproducible searches are conducted annually in the Embase, MEDLINE and PsycInfo databases. Retrieved records are screened against pre-defined eligibility criteria at title/abstract and full-text stages, and included studies are coded within the database to support searching. To 30 June 2020, over 430,000 records have been retrieved and screened, yielding nearly 4,800 trials and reviews for inclusion within the database. It has recently been used as a bibliographic database source in a number of scoping and systematic reviews (e.g., [30]). The OEF is presented as an online database publicly available for basic searching (https://www.orygen.org.au/Training/Evidence-Finder). However, for this review we had access to the backend database, which allowed us to construct a detailed and reproducible search strategy focused on peer support related terms (OEF backend database provided in Supplementary File 1). Table 2 lists the search terms applied to title, abstract, keyword, and label fields of each publication record within the OEF. The OEF currently contains studies published between 1980 and 30 June 2020, therefore, we undertook a second search process to retrieve studies published to 30 June 2021, and a third to update the search to retrieve studies published to 21 July 2022. This search strategy was conducted in the Embase, MEDLINE and PsycInfo databases (full search strategies available at SearchRxiv [31–33]). Finally, we conducted backward and forward reference searches through July 2022. Due to systematic searches returning few results, we additionally searched for existing youth peer support programs more broadly in July 2021 and again in July 2022 to check for any related research or evaluation reports; however, only one included study was found this way [34].

**Table 2:** Peer support search terms

#1 [peer*]
#2 [consumer OR patient OR service user OR survivor OR client]
#3 [operat* OR led OR run OR deliver* OR managed OR support* OR conducted OR assisted]
#4 [advoca*OR helper OR mentor OR leader OR counsel* OR educator OR aide OR consultant OR specialist OR train* OR advisor OR facilitat* OR provide*]
#5#2 AND #3
#6#2 AND #4
#7#1 OR #5 OR 6

### Eligibility criteria

We included studies that met the following inclusion criteria:

1. Mean age of participants between 14–24 years;
2. A controlled trial (either randomised or non-randomised);
3. The intervention involved provision of peer support by someone with lived experience of mental health challenges;
4. Reports at least one outcome measure related to depression or anxiety;
5. Full text available in English.

The age range reflected in the inclusion criteria was chosen, in line with the funder requirements and broader suite of work on active ingredients for youth anxiety and depression, based on the fact that symptoms of depression and anxiety most commonly emerge during this period, “reflecting a period of both vulnerability and opportunity for prevention and intervention” (page 6, [35]). A recent meta-analysis of 192 epidemiological studies demonstrated that the peak age of onset for mental disorders was 14.5 years and the median was 18 years, leading authors to call for a focus on “indicated, selective and/or universal preventive interventions for mental disorders during mid/late adolescence and young adulthood” (page 286, [2]). We excluded studies where the peer worker was not required to have lived experience due to the core values of peer support in the mental health context (i.e., as opposed to how the term peer support is used in an educational context) being focused on a sharing of lived experiences of mental health challenges and mental health service use [36].

### Study selection

*Covidence* was used to manage screening [37]; for the initial search, after removing duplicates, two reviewers (SC, EB) independently screened all titles/abstracts and resulting full texts, with disagreements resolved by a third reviewer (MBS). For the updated search (July 2021 to July 2022), three reviewers (EB, MBS, and BM listed in acknowledgements section) independently screened all titles/abstracts and resulting full texts, with disagreements resolved together by EB and MBS.

### Data extraction

Data were extracted from eligible studies into a standardised template (see Supplementary File 2) covering intervention details, attributes of peer workers, and outcomes measured. The data extraction form was developed by co-authors to include general items (e.g., design, control group) and items specific to peer support (e.g., details about the peer support intervention and peer worker). The steering group (see below for details) was consulted about any additional items to include in the form, and was amended accordingly (e.g., inclusion of detail about whether or not peer workers were matched to peers). A narrative summary of the findings was used to present the data outlined in the data extraction table. Studies were assessed for quality independently by SC and MBS using the Critical Appraisal Skills Programme (CASP; [38]; see Supplementary File 3).

### Registration

Time constraints prohibited us from registering our protocol before completing the review, as the work was undertaken through commission for a contract with a short duration and there were significant delays to protocol registrations being processed due to the COVID-19 global pandemic. We have nonetheless ensured reporting is in accordance with the Preferred Reporting Items for Systematic Reviews and Meta-Analyses (PRISMA) statement [39] (see Supplementary file 4 for checklist). We also opted for the pre-print to be published in ResearchSquare (doi.org/10.21203/rs.3.rs-1617867/v1 [40].

### Lived experience involvement and expert interviews

An experienced youth peer worker contributed to the initial proposal, but was unable to work on the review. An international steering group of youth peer workers and young people who had received peer support was established and convened by a lived experience expert (RM). The steering group consisted of 10 members aged 18–24, from four countries, including Australia (4), Canada (4), Ireland (1), and Singapore (1). The groups met for two hours fortnightly for the duration of the project. Discussion topics aligned with the stage of review (e.g., conceptualising peer support and determining search terms in earlier meetings, through to interpreting results and contextualising findings in later meetings). Additionally, interviews were conducted with nine experts from eight countries, including Australia, Brazil, Canada, India, Kenya, Nigeria, the United States, and Zambia. Interviewees were asked about any peer support programs they were aware of (to facilitate the broader searches for related research) and the relevance, appropriateness, and feasibility of peer support in their geographical region (to help inform the interpretation and discussion).

## Results

### Search results

**Fig. 1:**
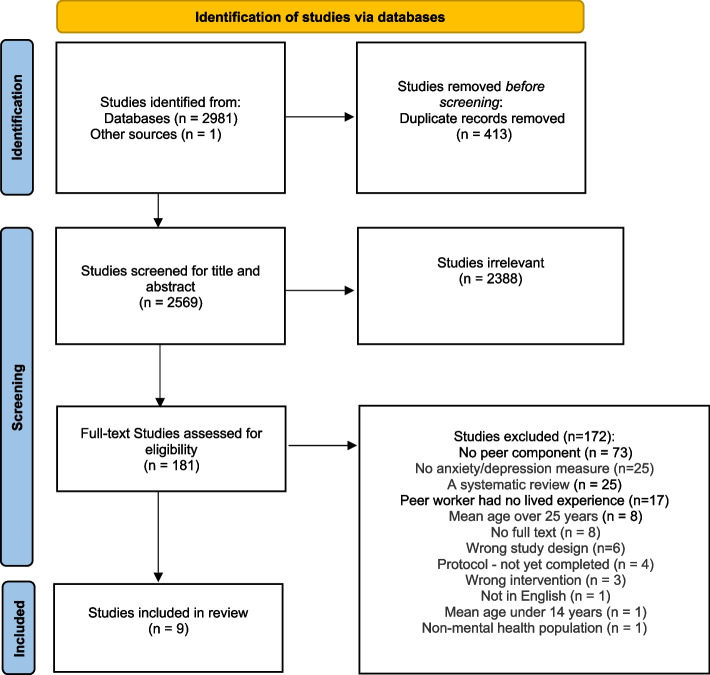
PRISMA flow diagram summarising study selection processes through the review

The searches retrieved 2,982 papers in total (see Fig. 1 for PRISMA flow diagram; [39]). Following removal of 413 duplicates, 2,569 papers were screened for eligibility. Of these, 2,388 were excluded following title and abstract screening, with a further 181 papers excluded after full text screening. Reasons for exclusion are reported in Fig. 1. In total, nine trials met inclusion criteria.

### Quality of studies

Supplementary File 3 shows the overall quality of the studies was good using the Critical Appraisal Skills Programme. No studies were excluded on the basis of quality. Importantly, seven studies were partially or fully unclear on blinding procedures. Four studies did not account for missing data and four studies did not conduct *a priori* power analyses. Intervention-specific fidelity (as opposed to more general measures of fidelity to the principles of peer support such as [41]) was assessed for two studies [34, 42] and was high for both.

### Study characteristics

Table 3 shows the characteristics of all nine studies, including sample, peer worker, and intervention characteristics. Of the nine studies identified, four were undertaken in North America, two in Oceania, and one each in Asia, Europe, and South America. Only one trial tested an intervention designed for anxiety and depression. The rest were designed for young people at risk of eating disorders or body image concerns (*n* = 4); any mental illness (*n* = 2), alcohol and drug misuse (*n* = 1), and first episode psychosis (*n* = 1). All nine studies were RCTs and all together included 2,003 participants. An overview of trial results is presented in Table 4, where available effect sizes were converted to cohen’s *d,* and if statistics for conversion were missing, original effect sizes from articles were reported. A full list of outcomes is listed in Supplementary File 5. Results are reported according to mental health conditions due to differences within peer support models used for different mental health conditions (e.g., group models more commonly used for eating disorders, digital interventions more common for mood disorders).

**Table 3:** Summary of study characteristic of included trials, including details of peer worker/s

Article	Country	N; gender	Mean Age (SD), years	Sample	Intervention/s Detail	Control	Peer-Worker	
							**Requirements**	**Training**
**Depression & Anxiety**								
Ellis et al. (2011)	Australia	39; 77% female, 23% male	19.67 (1.66); range 18–25	University Students experiencing low-to-moderate depression & anxiety	3 × 1-h sessions over 3 weeks working through the content of the MoodGarden website: a. Tools for self-management b. Bulletins on lifestyle management c. Discussions on effective treatments d. Messageboard for peer-based support e. Blogs and Charts	1. MoodGym Five modules of online CBT: a. Introduction to CBT b. Reducing dysfunctional thinking c. Overcoming negative feelings d. Identifying stress and relaxation e. Problem-solving strategies and enhancing relationships Control: No intervention	Lived experience with a mood disorder	Not reported
Conley et al. (2020)	USA	118; 82.2% female, 17.8% male	20.8 (4.99); range 18 or older	University Students who identify experiencing a mental health illness/ challenge	4 × 2–3-h sessions a. Consider the pros and cons of disclosing b. There are different ways to disclose c. Telling your story d. Booster Session: Check-in about whether participants have chosen to disclose	Control: Waitlist control	University Student with lived experience of a mental health illness or challenge	Peer facilitators completed two days of training in the HOP-C manual
Mulfinger et al. (2018)	Germany	98; 69% female, non-binary not reported	15.75 (1.15); range 13–18	Inpatients and outpatients with Anxiety and/or Depression	3 × 2-h weekly sessions over 3 weeks of Honest, Open, Proud (HOP): Themes: a. Challenging beliefs or self-stigma b. Pros & cons of disclosure c. The right person d. Telling one’s story e. Role of solidarity and peer support Workbooks contained vignettes, first-person accounts, worksheets, tables and role-plays 1 young expert and 1 young peer worker	Control: TAU	Young adult peer with lived experience of mental illness	Peer facilitators were trained by researchers and conducted a practice session
**First Episode Psychosis**								
Alvarez-Jimenez et al. (2021)	Australia	170; 47.1% female, 52.9% male	20.91 (2.88); range 16–27	Young people in recovery of First Episode Psychosis, exiting early intervention service	Mean number of individual posts and/or comments = 21.49 (SD = 41.71) over 18 months (Mean = 8.15 (SD = 5.65)) of Online Social Network: a. Icebreakers b. user-generated threads c. content related to mental health d. content of general interest Vocational training: a. Ask the expert b. individualized online vocational support Online Therapy w/ health professional: a. Online pathways – distinct themes on recovery b. Online Steps—interactive therapy modules	Control: TAU	Peer workers had lived experience of mental illness	Not reported
**Prevention of Eating Disorders**								
Becker et al. (2010)	USA	102; 100% female	18.73 (0.72); range 18–21	University students (Freshman or Sophomore)	2 × 2-h sessions over 2 weeks of Cognitive Dissonance: a. Group discussions on the thin ideal b. Group brainstorming on cost of the thin ideal c. Homework body appreciation d. Role-plays on resisting the thin ideal e. Shared lived experiences from peer-leaders and peers in the sessions	1.Media Advocacy: a. Watch videos b. Group discussions of the thin ideal c. Group brainstorming on cost of the thin ideal d. Food and exercise diaries	Peer-leaders with past participation in the program and a member or the sorority	4.5 h experiential training sessions. Peer-leaders trained in teams (3 – 4 peer-leaders at a time)
Ciao et al. (2021)	USA	Trial 1 *N* = 98; female 80% male 14% non-binary 6% Trial 2 *N* = 141; 80% female 15% male 5% non-binary	Trial 1 20.39 (4.12); range 18–50 Trial 2 19.66 (2.53); Range 18–36	University students interested in a body acceptance program	2 × 2-h group sessions over 2 weeks of the ‘EVERYbody Project’ involving 4–9 participants a. Group discussions b. Role-play c. Group activities Trial 1: 1 × Expert-leader & 2 x peer-leaders Trial 2: 3–4 × peer-leaders	Trial 1: Control: No intervention Trial 2: Video intervention a. Watch videos b. Reflective writing	Peer-leaders with past participation in the female program	Training followed protocol used in the peer-led ‘Body Project’ Trial 1 2 days of training (16 h) Trial 2 2 days of training (16 h) with discussion on group facilitation skills and role plays on difficult diverse situations
Kilpela et al. (2016)	USA	180; 62% female, 38% male	19.9 (1.2); range 18–23	University students	2 × 2-h sessions over 2 weeks of ‘Body Project’ a. Group discussions on the thin ideal contrasting healthy ideals, the origin of the thin ideal, past pressures b. Group brainstorming on cost of the thin ideal and combating the thin ideal c. Homework: body appreciation, writing to a young person, challenging own behaviour d. Role-plays on resisting the thin ideal e. Shared lived experiences from peer leaders and peers in the sessions	Control: Waitlist	Peer-leaders with past participation in the program	Trainer to Trainer method: Experienced peer leaders in the program trained new peer leaders over two days
Resendel et al. (2021)	Brazil	74; 100% female	20.5 (2.02); range 18–30	University students	4 × 60-min sessions over 4 weeks of ‘Body Project’ led by two peer leaders a. Group discussions on the thin ideal, body appreciation/concerns b. Group brainstorming on the cost of the thin ideal c. Homework sessions on body appreciation d. Role-plays on resisting the thin ideal e. Engaged in further body activism	Control: No intervention	Have experience in Intuitive Eating and past body image concerns and currently a university student	References the Body Project website: Therefore, training on the concept and rationale of the body project, in group discussions and role plays, using the manual for guidance. Possibly some practice runs with feedback from supervisors, but unclear from publication
**Prevention in Substance Use**								
German et al. (2012)	Thailand	983; 27.3% female 72.7% male	Median 19 (IQR = 18–20); range 18–25	Used meth-amphetamine & engaged in sex at least 3 times in the last 3 months	7 × 1.5–2.5 h group session twice weekly over 1 month of ‘Peer Education’, in groups of 8–12 participants: a. Drug use on individuals b. Drug use and social influences c. Drug use and sex d. Drug use and risk behaviours e. Family and community f. Community project g. Review and graduation 2 boosters (3 & 6-month mark)	7 × 1.5–2.5 h group session twice weekly over 1 month of ‘Life Skills’, in groups of 8–12 participants: a. Understanding life b. Decision making skills and old friends/new friends c. Danger of drug use d. Sexually infectious diseases e. How important is stress? f. Emotion management and life goals g. Envelope of goodness and graduation Network groups for participants in both Peer Education and Life Skills received no intervention	Early 20 s, participated in an earlier study as part of the ethnography team	Researchers trained peer facilitators in a 1 week long intensive training session on building a prosocial role and to increase positive communication and interactions with peers

**Table 4:** Summary results of anxiety and depression outcomes of included trials

	Ellis et al. (2011)	Conley et al. (2020)	Mulfinger et al. (2018)	Alvarez-Jimenez et al. (2021)	Becker et al. (2010)	Ciao et al. (2021)	Kipela et al. (2016)	Resende et al. (2021)	German et al. (2012)
Measures	Anxiety and Depression (DASS-21);	Anxiety (GAD-7) Depression (CESD)	Depression (CESD)	Depression (CDSS)	Negative affect (PANAS-X)	Negative Affect (PANAS)	Negative Affect (PANAS)	Negative Affect (PANAS-B)	Depression (CESD)
Groups	Online peer support (*n* = 13) Online CBT (*n* = 13) Controls (*n* = 13)	HOP-C (*n* = 63) Control (*n* = 55)	HOP (*n* = 49) Control (TAU; *n* = 49)	Horyzons + TAU (*n* = 84) Control (TAU; *n* = 86)	Cognitive dissonance (*n* = 53) Control (Media advocacy; *n* = 49)	Trial 1: Everybody Project (w/ expert—and peer leaders; *n* = 48) Control (waitlist; *n* = 50) Trial 2: Everybody Project (w/ peer leaders only; *n* = 65) Control (Video intervention; *n* = 76)	Mixed-gender (*n* = 77, Female-only (*n* = 65) Control (waitlist; *n* = 38)	Body Project (*n* = 38) Control (*n* = 36)	Peer education (*n* = 209) Control (Network peer education; *n* = 286) Life Skills (*n* = 206) Control (Network life skills; *n* = 282)
**Anxiety and Depression Results**									
Anxiety	Y*; online CBT, *d* = 0.99, *p* = .03, and online peer support, *d* = 0.95, *p* = .01, compared to controls	ns							
Depression	ns	ns	ns/Y* FU6W; HOP**,** *d* = 0.72, *p* < .001) compared to controls	ns					Y* Intervention: post-intervention mean difference = − 4.5251, *SE* = 0.7279; *p* < 0.0001), compared to baseline Control: ns
Negative Affect					Y* Intervention: post-intervention, *d* = 0.51, *p* < .05; 8 weeks *d* = 0.25, *p* < .05; 8 months *d* = 0.35, *p* < . 05; 14 months *d* = 0.48, *p* < .05, compared to baseline Control: post-intervention, ns; 8 weeks, ns; 8 months *d* = 0.58, *p* < .05; 14 months *d* = 0.34, *p* < .05, compared to baseline	Y* Trial 1 EVERYbody (w/ expert and peerleaders): post-intervention *d* = 0.56, *p* < .05; 1-month *d* = 0.42, *p* < .05, compared to controls; group x time interaction (*b* = − 0.03, *t* = − 4.45, *p* < .0001) Trial 2 EVERYbody (w/ peer-leaders only): post-intervention *d* = 0.01, *p* < .05; 1-month *d* = 0.10, *p* < .05 compared to controls; group x time interaction, ns	Y*^a^ Males Intervention: Males in mixed gender group: postintervention, *d* = 0.40, *p* = 0.0080; 2-month ns; 6-month ns, compared to controls Females in mixed gender group: ns Females intervention: Female only group: ns	ns/Y*^b^** FU24W** Intervention: *d* = 0.60 *p* < .05) compared to controls	
Participant Feedback	Online peer support compared to online CBT: • More helpful, • More enjoyable • Equally recommended • One-third reported they would continue using online peer support • Two-thirds reported they would continue using online CBT – possibly because the online CBT group were still feeling more anxious than the online peer-support group	Not reported	• Peer leader was viewed as an inspiring role-model • Enjoyed learning about their real-life experiences • Relief to talk about disclosure in a safe space • facilitated openness, trust, and respect within the group interactions • Some materials were deemed too theoretical, demanding, and hard to concentrate on or too detailed	• Some participants had positive experiences of social connection on Horyzons • Others did not due to social anxiety, paranoia and confusion within the social network of Horyzons	• Participants preferred the cognitive dissonance intervention over the control intervention • Both interventions were deemed useful • Suggested the control intervention may be a good follow up or refresher intervention	• Experts were able to address diverse body images (various gender, sexual, and racial identities) better than peer leaders • Peer leaders lacked lived experience in all diverse body images, which may have hindered them in connecting with all the participants	• Adding males to the Body Project created a mixed gender group, which established a warmer collaborative (versus activating) vibe • Normally the Body Project creates an angry vibe against the thin ideal through the historical struggle women have had against the thin ideal • This warmer vibe was carried over to the female only group, which may explain a lack of effects among females	Not reported	The research site (known as “House of Friends”): • Allowed participants to gather and socialise informally with each other • Was a safe place, as there was no social stigma or fear of being arrested, which was common for participants in the general community

*Note:* Y*(bolded) = significant result, *ns* non-significant, *FU* Follow-up, *(number)W/M* number of weeks or months, *Note*: *DASS-21* Depression, Anxiety and Stress Scale, *GAD-7* Generalized Anxiety Disorder 7-item Scale, *CESD* Center for Epidemiologic Studies Depression Scale, *CDSS* Calgary Depression Scale for Schizophrenia, *PANSS* Positive and Negative Syndrome Scale, *PANASX* Positive and Negative Affect Schedule – Revised, *PANAS* Positive and Negative Affect Schedule, *PANAS-B* Positive and Negative Affect Schedule

a Kipela et al. (2016) found negative affect only decreased with the males in their mixed-gender group, not with the females in the mixed-gender group or with the female only group

b Resende et al. (2021) found negative affect only decreased at the 24-week follow-up, not at post-intervention, or four weeks follow-up

### Depression and anxiety

The first study on depression and anxiety was undertaken in Australia with university students experiencing low-to-moderate depression & anxiety [43]. Students were randomised into one of two experimental groups or a no-intervention control group. The first group engaged in online CBT via ‘MoodGym’ and the second group engaged in an online peer support group via ‘MoodGarden’ (see Table 3 for intervention characteristics). ‘MoodGarden’ involved access to the established non-profit website run by volunteers with lived experience of a mood disorder. Peer workers mediated the message board that the participants posted on. Compared to the control group, post-intervention measures showed that the online CBT and online peer support significantly reduced anxiety symptoms (see Table 4). However, neither intervention affected depression symptoms. ‘MoodGarden’ participants reported higher perceived online social support compared to the ‘MoodGym’ and control groups (see Supplementary File 5).

### Any mental illness

The second study [44] was undertaken in Germany with teenagers (mean age 16) experiencing depression and anxiety, mainly in inpatient services. They compared an intervention known as Honest, Open, Proud (HOP) to a treatment as usual (TAU) control group (see Table 3 for intervention details). This group program was co-facilitated by a peer worker and aimed to support individuals in their decisions to disclose their mental illness and therefore reduce the impact of stigma for adolescents. Depressive symptoms were measured as secondary outcomes and showed no reduction post-intervention; however, at six-week follow-up, depressive symptoms had significantly reduced (see Table 4). There was a significant difference between groups in favour of the intervention group for the primary endpoints (reduction in stigma stress post-intervention and improvement in quality of life at follow up). Effects identified which may overlap with peer support mechanisms included help-seeking intentions (family/friends, professionals), stage of recovery, and empowerment (self-esteem, optimism; see Supplementary File 5). No effect was found for social withdrawal or hopelessness. Attrition rates between post-intervention and follow-up were the same for both groups (*n* = 11); reasons included being uncontactable or refusing to complete follow-up.

This study was the only included study to conduct a cost-effectiveness analysis aimed at calculating value for money of delivery of the intervention. HOP’s total costs, which, for example, included training and employment of peers and professionals, as well as overhead costs, were calculated and compared to British annual costs per young person (aged 5 to 15) where the National Institute for Health and Care Excellence (NICE) uses a cost-effectiveness threshold of £20,000–£30,000/quality-adjusted life year (QALY) [45]. Based on the utility gains (0.044), HOPS was deemed to be a cost-effective intervention, even if those gains only lasted for two months (at a cost of €20,533/QALY), but more so if the gains continued at six months (€6,969/QALY).

A separate trial tested an adapted version of HOP (called Honest Open Proud – College, or HOP-C) for tertiary students in two urban areas of the United States [34]. The intervention had been adapted prior to the trial using community based participatory research methods for use in College settings. Although the core elements of the intervention remained the same as the HOP model described aboves, changes included: 1) a shift in focus from severe mental illness to depression and anxiety; 2) the revision of course materials, including vignettes, to include reference to college staff and peers; and 3) an additional section focusing on disclosure through social media. Participants (*n* = 118) were randomised to receive HOP-C (*n* = 63) or a waitlist control (*n* = 55). Depression and anxiety were measured as secondary, exploratory outcomes at pre-intervention, post-intervention, and after the booster session (2–3 weeks later). Whilst there were significant effects for stigma related outcomes favouring the intervention group, there was no significant effects for either depression (*p* = 0.74) or anxiety (*p* = 0.21).

### First episode psychosis

One study that assessed depression in young people with a recent onset psychotic disorder who had been discharged from a specialist early intervention service in Australia was identified [46]. The intervention ‘Horyzons’ used the ‘Moderated Online Social Therapy’ model, which integrated interactive online therapy, peer-to-peer online social networking, peer moderation, and expert support (see Table 3). While the intervention was shown to improve vocational functioning and reduce hospital emergency service use compared to TAU, no effect was found for depression symptoms (*p* = 0.42) or any peer support related constructs over the 18-month follow-up period (e.g. loneliness; see Supplementary File 5).

### Eating disorders

Four studies tested slightly different versions of a lived-experience peer worker-led intervention known as the Body Project Collaborative (see Table 3 for intervention details), which aims to prevent eating disorders and body dissatisfaction. All studies were successful in reducing body image concerns and eating disorder risk. All involved university students, three in the USA [47–49], and one in Brazil [50]. All utilised the Positive and Negative Affect Schedule, with the effect of these interventions on negative affect varying across trials. Table 4 shows two [47, 48] found significant reduction of negative affect over time. Resende and colleagues [50] found an increase in self-esteem at post-intervention and at 24-week follow-up (see Supplementary File 5). However, they only found a significant reduction in negative affect at 24 weeks. Kilpela, Blomquist [49] reported a significant reduction of negative affect only in male participants. Overall, peer worker-led interventions across studies had significantly better outcomes compared to control groups (which included waitlist controls, a video and expressive writing condition, and an assessment-only control condition; see Table 3).

### Substance use

The last of the included studies evaluated an intervention for young methamphetamine users in Thailand [51], which was compared with an active control group. Secondary analysis of the trial demonstrated a significant effect for depression symptoms; however, this was not the primary aim of the trial. The intervention was a ‘Peer Education’ group that aimed to teach participants to reduce their methamphetamine use and sexual risk behaviours as well as how to communicate learnings from the group with their methamphetamine using peers or sexual partners. The authors hypothesised the intervention had a significant impact on depression (see Table 4) due to the intervention encouraging participants to build a prosocial role and increase positive communication with peers and family members. The emphasis on social relationships and contributing to the community may have affected feelings of isolation and stigma, particularly within a collectivist culture such as Thailand.

## Discussion

### Overall findings

In this review, we aimed to identify and describe studies of peer support for young people to improve symptoms of depression or anxiety. We sought to investigate in which ways, in which contexts, and for whom, peer support appears to work or not work. Despite there being a range of controlled trials testing peer support interventions for adults with mental health challenges such as anxiety and depression [52], very few have been conducted in young people. We were only able to identify two trials specifically targeting anxiety or depression, and there were limitations across studies that we will now discuss.

In total, six studies were conducted in high income countries and two in low- and middle-income countries. The most common setting was universities (6 studies), with only two mental health service settings and one community research centre. Aside from the considerable lack of geographical diversity, the settings also limit our knowledge of the context in which peer support might work, given that only some young people attend mental health services and university. Many studies in a broader range of settings were excluded because the peer worker role did not require lived experience. The variability of this requirement, which is mirrored in research with adults [15–17], is just one factor that is indicative of the generally heterogeneous array of definitions of peer support, in terms of the setting, intervention, and characteristics of the peer worker.

There are also limitations specific to the review design and methodology. The age range of interest in this review (i.e., 14–24 years), is not often reflected in study inclusion criteria. Therefore, we made the pragmatic decision to include studies if the *mean* age of participants fell in the age range of interest. Based on the age ranges and mean ages for each study, some of the participants in the included trials could have been older than 24; however, based on the low standard deviations, it is likely that only a very small, negligible number of participants would have been older than 24. Although the Orygen Evidence Finder (OEF) includes studies retrieved from the key databases we would have targeted in a review not using the OEF (i.e., PsycInfo, MEDLINE and Embase), the OEF team manually screen studies for inclusion. This means that due to human error some studies may be missed, and these omissions would also be reflected in our searches. A further limitation is that we did not prospectively register the review protocol, meaning that the opportunity to reduce bias and duplication, and improve transparency was lost [53]. Lastly, due to the heterogeneity of the studies, we did not undertake a meta-analysis or other type of formal synthesis. Our hope is that as the field develops further and there is improved consistency in both approaches (e.g., outcomes) and reporting that this will be possible.

### In what contexts does peer support work?

Importantly, two studies [43, 46] successfully and safely tested online peer support interventions. Understanding how peer support can be delivered remotely is important in the context of the current COVID-19 global pandemic, and complements work done on peer-to-peer support [54]. As young people globally grapple with the increase in social isolation, uncertainty about the future, and other experiences that are related to poorer mental health, having sufficient workforce supply to serve the demand of those seeking help is essential. Although they should not be seen as ‘cheap labour’, with the right support structures in place [26], peer workers are able to be trained more readily than other professions. One study specifically assessed and reported no adverse effects [47]; participant feedback from the other studies was generally good (see Table 4) and successful interventions were found to be acceptable, suitable, and cost-effective, in line with adult reviews [15, 17, 20]. It is also easier to ensure diversity in the workforce through peer support, as the barriers to formal education pathways that exist for many minority groups are less prominent in peer work. As our steering group members pointed out, better representation from minority groups in the workforce is more likely to result in culturally safer environments.

### For whom does peer support work?

In terms of understanding who peer support may or may not work for, we were unable to answer this. Only two studies focused specifically on depression and anxiety, with the rest focused on general mental health challenges, relapse prevention after first episode psychosis, substance use disorders and four focused on the prevention of eating disorders. In line with adult literature, depression-related measures were more commonly used and anxiety-related measures were largely absent [52]. Unlike the adult peer support literature as reviewed by King, Simmons [15], the studies we found in youth settings did not aim to measure the impact of peer support; any overlapping measures such as hope and empowerment were tied back to their initial study aims rather than peer support components.

Further, the mean age of participants in all but one trial fell in the young adult range (19–21 years). Peer support has been widely tested in educational settings with children and adolescents; however, peers are not required to have lived experience, meaning they don’t contribute to our understanding of lived experience as an active ingredient or align with the core principles of peer support in mental health contexts. However, several studies were conducted in university settings with young adult-aged peer workers who had lived experience of mental health challenges. Five included studies found positive outcomes (and no detriment) in this setting, including reduced anxiety, reduced affect, increased self-esteem, and higher perceived online social support, suggesting that peer work can be effective within a university environment for young adults. Though, developing and testing lived experience-based peer support in younger groups requires careful consideration of what age one might expect a peer worker to be [55, 56]. While having a peer worker be as close in age as possible, it also makes good sense to have slightly older peer workers who have experienced both relevant mental health challenges and some experiences of treatment and recovery. Overall, it is not yet discernible who peer support does and does not work for.

### Why might peer support work?

Specific mechanisms of action for peer support in youth depression and anxiety are yet to be proposed and tested. The lack of clarity in the evidence we did find (e.g. definition of a peer worker, what the intervention is, and what peer support values or principles were adhered to) makes it even more difficult to understand mechanisms. Consequently, empirical work that tests this proposed model is required.

Other critical gaps in the literature include exploring the best ways to test the ‘effectiveness’ of peer support interventions in this area. When our systematic review failed to identify many studies, we searched for existing youth peer support programs and checked for any related research or evaluation. We found a number of programs operating in a range of countries, yet we did not find any associated evaluations, suggesting a lost opportunity to properly understand how these programs are helping young people who experience depression and anxiety. In contrast, the steering groups responded optimistically, suggesting there is a wealth of knowledge to draw on from the programs run by groups who are out helping young people 'on the ground'.

Harnessing this knowledge will require careful consideration of what types of research designs and methodologies are appropriate for peer support [57]. Much of mental health research is based on a medical/clinical model that focuses on individual deficits [58]. This is at odds with both the theoretical underpinnings of peer support models (e.g. Intentional Peer Support) [57] and also the collectivist nature of many cultures worldwide, as recognised by one of the trials identified in our review [51]. Focusing on the programs already operating, mainly in high income countries, is essential in order to capture the lessons already learned about how people with lived experience of anxiety and depression can help their peers. Understanding how peer support programs operate effectively in low-resource settings and in varying cultural contexts, yet retain relevant core values of peer support where appropriate, is equally as important. Furthermore, peer support has also been associated with engaging in generative actions such as helping others, changing organisations and systems, and sharing personal stories [59], which can lead to a range of psychosocial benefits at both the individual and relational interpersonal levels [10, 18].

Regardless of the research setting and cultural context, this review highlighted a number of areas requiring clarity. First of all, role-specific definitions of *peer* and *lived experience* are vital, including relevancy of age, characteristics and type of lived experience (e.g. experience of mental health challenges, receipt of treatment, and recovery). Secondly, detailed descriptions of peer support interventions are required that explain: 1) the theoretical underpinning, core values and principles of the intervention; 2) how fidelity to is assessed; 3) the nature of the role and how the peer workers were supported to adhere to these values and principles in their role (i.e. training and supervision). Further, given that a number of barriers to implementing peer work in practice have been identified for adults (e.g. [13]) and are likely exacerbated for young people [55, 56], future research should use designs incorporating implementation science methodologies from the outset. As peer work is increasingly becoming a focus of research, it is important that knowledge is well translated into program implementation to ensure that youth peer workers are supported and programs are cost-effective and adequately integrated into existing services. Implementation science approaches that take into account lived experience and peer worker perspectives (e.g., [60]) are needed to further investigate how contextual factors influence successful program operation, and to develop and apply strategies to address barriers. There are also unresolved issues beyond the scope of research, such as what happens to youth peer workers when they age out of the age-related role requirements. All of these elements were generally lacking in the literature we reviewed, but are critical for moving the field forward.

Lastly, only two of the studies used a co-design approach to intervention development. These interventions were both digital in nature, which is unsurprising given that user-centred design methodologies are common in the development of digital solutions. Involving young people with lived experience of anxiety and depression in all aspects of peer support intervention design and testing will improve the quality and significance of such endeavours [61]. Similarly, involvement from experienced youth peer workers will also help ensure interventions are appropriate, feasible, and meaningful. Drawing on the existing knowledge held by the youth peer workforce and young people who have accessed peer support interventions is the most promising avenue for determining the ways in which peer support is an active ingredient for youth depression and anxiety.

## Supplementary Information

**Additional file 1. **Orygen Evidence Finder backend database.

**Additional file 2. **Data extraction template.

**Additional file 3. **Critical Appraisal Skills Programme ratings.

**Additional file 4. **PRISMA checklist.

**Additional file 5. **Additional outcomes reported by included trials.

## Data Availability

The datasets used and/or analysed during the current study are available from the corresponding author on reasonable request.

## Abbreviations

CASP: Critical Appraisal Skills Programme
CBT: Cognitive Behavioural Therapy
HOP: Honest, Open, Proud
NICE: The National Institute for Health and Care Excellence
OEF: Orygen Evidence Finder
PRISMA: Preferred Reporting Items for Systematic Reviews and Meta-Analyses
QALY: Quality adjusted life years
RCT: Randomised controlled trial
SES: Socio-economic status
TAU: Treatment as usual
USA: United States of America
