# Supplementary material for: The effectiveness of peer support from a person with lived experience of mental health challenges for young people with anxiety and depression: a systematic review
Source: BMC Psychiatry. 2023 Mar 24;23:194. doi: 10.1186/s12888-023-04578-2 (PMC10038377; doi:10.1186/s12888-023-04578-2)
Supplement: Supplementary file 2 — Additional file 2. Data extraction template. [file 12888_2023_4578_MOESM2_ESM.docx]

| **Study** | | | | | **Sample Characteristics** | | | | | | **Peer Support Program** | | | | | | **Peer Support Worker** | | | | **Study Outcomes** | | | | |
| --- | --- | --- | --- | --- | --- | --- | --- | --- | --- | --- | --- | --- | --- | --- | --- | --- | --- | --- | --- | --- | --- | --- | --- | --- | --- |
| Author | Year | Title | doi | Country | N | Diagnosis/ Complaint/ Symptoms | Age M (SD) | % females | Participant Origin (outpatient, community, exiting inpatient) | Control Group Matched/Unmatched (details) | Setting (type of mental health service, educational institution, online, integrated, standalone) | Peer Support Model (designed deliverables) | Overall Length of Time | Frequency and Time Length Delivered | Individual/Group | Structured/ Unstructured | Peer Worker Description/ Requirements (lived experience, age, gender, matched to client?) | Peer Worker's Duties | Training | Supervision/Support | Study Design | Outcomes measured | Summary of key findings | Notes (eg. Are outcomes related to designed deliverables) |  |
