# Supplementary material for: The effectiveness of peer support from a person with lived experience of mental health challenges for young people with anxiety and depression: a systematic review
Source: BMC Psychiatry. 2023 Mar 24;23:194. doi: 10.1186/s12888-023-04578-2 (PMC10038377; doi:10.1186/s12888-023-04578-2)
Supplement: Supplementary file 3 — Additional file 3. Critical Appraisal Skills Programme ratings. [file 12888_2023_4578_MOESM3_ESM.docx]

**Supplementary File 3:** Critical Appraisal Skills Programme ratings.

|  | Alvarez-Jimenez et al. (2021) | Becker et al. (2010) | Conley et al. (2020) | Ciao et al. (2021) | Ellis et al. (2011) | German et al. (2012) | Kipela et al. (2016) | Mulfinger et al. (2018) | Resende et al. (2021) |
| --- | --- | --- | --- | --- | --- | --- | --- | --- | --- |
|  | Section A: Is the basic study design valid for a randomised controlled trial? | | | | | | | | |
| Did the study address a clearly focused research question? | Yes | Yes | Yes | Yes | Yes | Yes | Yes | Yes | Yes |
| Was the assignment of participants to interventions randomised? | Yes | Yes | Yes | Yes | Yes | Yes | Yes | Yes | Yes |
| Were all participants who entered the study  accounted for at its conclusion? | Yes | Yes | Yes | Yes | Yes | Yes | Yes | Yes | Yes |
|  | Section B: Was the study methodologically sound? | | | | | | | | |
| Were the participants ‘blind’ to  intervention they were given? | Yes | Yes | No | Unclear | Unclear | Unclear | Unclear | No | Unclear |
| Were the investigators ‘blind’ to the  intervention they were giving to  participants? | No | Unclear | No | Unclear | Unclear | No | No | No | Unclear |
| Were the people assessing/analysing  outcome/s ‘blinded’? | Yes | Yes | No | Unclear | Unclear | Yes | No | No | Unclear |
| Were the study groups similar at the start of  the randomised controlled trial? | Yes | Yes | Yes | Yes | Yes | Yes | Yes | Yes | Yes |
| Apart from the experimental intervention, did  each study group receive the same level of  care (that is, were they treated equally)? | Yes | Yes | Yes | Yes | Yes | Yes | Yes | Yes | Yes |
|  | Section C: What are the results? | | | | | | | | |
| Were the effects of intervention reported  comprehensively? | Yes | Yes^#^ | Yes | Yes^# (trial 1)^ | Yes^*#^ | Yes^*#^ | Yes^*^ | Yes | Yes^*^ |
| Was the precision of the estimate of the intervention or treatment effect reported? | Yes | No | Yes | Yes | No | Yes | No | Yes | Yes |
| Do the benefits of the experimental  intervention outweigh the harms and costs? | No (non  Significant  effect) | Yes | Yes | Yes | Yes | Unclear | Unclear | Yes | Yes |
|  | Section D: Will the results help locally? | | | | | | | | |
| Can the results be applied to your local population/in your context? | Unclear | Unclear | Unclear | Unclear | Unclear | Unclear | Unclear | Unclear | Unclear |
| Would the experimental intervention provide greater value to the people in your care than  any of the existing interventions? | Unclear | Unclear | Unclear | Unclear | Unclear | Unclear | Unclear | Unclear | Unclear |

*Note:* ^*^= did not account for missing data; ^#^ = did not conduct *a prior* power analysis
