# Supplementary material for: The effectiveness of peer support from a person with lived experience of mental health challenges for young people with anxiety and depression: a systematic review
Source: BMC Psychiatry. 2023 Mar 24;23:194. doi: 10.1186/s12888-023-04578-2 (PMC10038377; doi:10.1186/s12888-023-04578-2)
Supplement: Supplementary file 5 — Additional file 5. Additional outcomes reported by included trials. [file 12888_2023_4578_MOESM5_ESM.docx]

**Supplementary file 5:** Additional outcomes reported by included trials

|  | Ellis et al. (2011) | Mulfinger et al. (2018) | Alvarez-Jimenez et al. (2021) | Becker et al. (2010) | Ciao et al. (2021) | Kipela et al. (2016) | Resende et al. (2021) | German et al. (2012) | Conley et al (2020) |
| --- | --- | --- | --- | --- | --- | --- | --- | --- | --- |
| Measures | Anxiety and Depression (DASS-21);  Social Support (OSSS);  CBT Literacy (CBT-Lit);  Automatic Thoughts (ATQ) | Depression (CESD)  Empowerment (ES)  Social Functioning (SCOS)  Help-Seeking (GHSQ)  Quality of Life (QoL)  Stage of Recovery (SISRS)  Stigma Stress (SSS)  Self-Stigma ((ISMI & SSMIS)  Disclosure related Distress (DD)  Hope BHS  Secrecy SCOS  Attitudes to disclosure (AD) | Depression (CDSS)  Social Functioning (PSP)  Social Support (MOS-SSS)  Self-Esteem (SERS-SF)  Quality of Life (AQoL-8D)  Loneliness (UCLA)  Self-Efficacy (MHCS)  Satisfaction with Life (SWLS)  Positive Symptoms of psychosis (PANSS Subscale Positive)  Negative Symptoms of psychosis (PANSS subscale Negative) | Negative affect (PANAS-X)  Thin-ideal Internalization IBSS-R  Body Dissatisfaction (EDE-Q)  Bulimic Pathology (EDE-Q bulimic scale)  Eating Disorder Pathology (EDE-Q restraint subscale) | Negative Affect (PANAS)  Thin-ideal Internalization (SATAQ-4R -INT)  Body Dissatisfaction SDBPS  Eating Disorder Pathology EDE-Q | Negative Affect (PANAS)  Thin-ideal Internalization (SATAQ-4)  Body Dissatisfaction (BPSS-R)  Eating Disorder Pathology (EDE-Q2)  Male Body Attitudes (MBAS) | Negative Affect (PANAS-B)  Self-Esteem (RSES)  Thin-ideal Internalization (SATAQ-4R)  Body Shape (BSQ-8)  Eating Attitudes (EAT-26)  Intuitive Eating (IES-2)  Body Appreciation (BAS-2) | Depression (CESD)  Meth-amphetamine Used  Condom Used | Depression (CES-D 10)  Anxiety (GAD-7)  Self-stigma (SSMIS-SF)  Stigma Stress (SSS)  Self-efficacy about disclosure or secrecy |
| Groups | Online Peer Support (n = 13)  Online CBT (n = 13)  Controls (n =13) | HOP (n = 49)  Controls (TAU; n = 49) | Horyzons + TAU (n = 84)  Control (TAU;  n = 86) | Cognitive Dissonance (n = 53) Control (Media Advocacy; n = 49) | Trial 1: Everybody Project (w/ expert - and peer-leaders; n = 48)  Control (Waitlist; n = 50)  Trial 2: Everybody Project (w/ peer-leaders only; n = 65)  Control (Video Intervention; n = 76) | Mixed-Gender (n = 77,  Female-Only (n = 65)  Control (waitlist;  n = 38) | Body Project (n = 38)  Control (n = 36) | Peer Education  (n = 209)  Control (Network Peer Education;  n = 286)  Life Skills  (n = 206)  Control (Network Life Skills;  n = 282) | Honest, Open, Proud–College (HOP-C) (n=63)  Waitlist control (n=55) |
| **Peer Support Related Mechanisms Results** | | | | | | | | |  |
| Empowerment |  | ns/**Y* FU6W** HOP**,** subscale self-esteem, postintervention, ns; 6-weeks, *d* = 0.26, *p* = .09; subscale optimism, postintervention, ns; 6-weeks *d* = 0.35, *p* = .09 compared to controls. |  |  |  |  |  |  |  |
| Hope |  | ns |  |  |  |  |  |  |  |
| Social Functioning |  | ns | ns |  |  |  |  |  |  |
| Social Support | **Y*;** online social support group compared to controls, *d* = 0.83,  *p*= .00 and online CBT group, *d* = 0.59, *p* = .00. |  | ns |  |  |  |  |  |  |
| Help-Seeking |  | **Y*** HOP subscale family/friends, postintervention, d = 0.55, p < .001; 6 weeks, ns, subscale professional**,** postintervention, d = 0.47, p = .010; 6 weeks, d = 0.64, p = .002 compared to controls. |  |  |  |  |  |  |  |
| Self-Esteem |  |  | ns |  |  |  | **Y*** Intervention: postintervention *d* = 0.98 *p* < 0.001; 4-weeks *d* = 1.87 *p* < 0.001; 24 weeks *d* = 1.38 *p* < 0.001, compared to controls |  |  |
| Quality of Life |  | ns/**Y* FU6W** HOP postintervention, ns; 6-weeks, *d* = 0.60, *p* = .004 compared to controls. | ns |  |  |  |  |  |  |
| Stage of Recovery |  | ns/**Y* FU6W** HOP (postintervention, ns; 6 weeks *d* = .35, *p* = .037) compared to controls. |  |  |  |  |  |  |  |
| Loneliness |  |  | ns |  |  |  |  |  |  |
| Self-Efficacy |  |  | ns |  |  |  |  |  | Self-efficacy disclosing HOP (post booster session d = .10, p<.003 (Bonferroni correction)) |
| Satisfaction with life |  |  | ns |  |  |  |  |  |  |
| **Results Specifically Related to Study Aims** | | | | | | | | |  |
| CBT Literacy | **Y*** NPS; CBT online compared to controls *d* = 0.52, *p* = .02, and online peer support, ns. |  |  |  |  |  |  |  |  |
| Automatic Thoughts | ns |  |  |  |  |  |  |  |  |
| Stigma Stress |  | **Y*** HOP postintervention, *d* = 0.92, *p* < .001; 6 weeks, *d* = 0.96, *p* < .001 compared to controls. |  |  |  |  |  |  |  |
| Self-Stigma |  | **Y*** HOP subscale internalised**,** postintervention, ns; 6 weeks, *d* = 0.61, *p* < .001; subscale negative stereotyping, postintervention, *d* = 0.36, *p* =.018; 6 weeks, *d* = 0.63, *p* = .001 compared to controls. |  |  |  |  |  |  | ns |
| Disclosure related Distress |  | **Y*** HOP, postintervention, *d* = 0.53, *p* < .001; 6 weeks, *d* =0.71, *p* < .001 compared to controls. |  |  |  |  |  |  |  |
| Secrecy |  | ns/**Y* FU6W** HOP**,** postintervention, ns; 6 weeks, *d* =0.81, *p* < .001 compared to controls. |  |  |  |  |  |  |  |
| Attitudes to disclosure |  | **Y*** HOP**,** subscale family/friends, postintervention, *d* = 0.62 *p* < .001; 6 weeks, *d* = 0.64, *p* = 0.001; subscale teacher/employer, postintervention, *d* = .46, *p* = .011; 6 weeks, *d* = .64, *p* = .005) compared to controls. |  |  |  |  |  |  |  |
| Positive Symptoms of Psychosis |  |  | ns |  |  |  |  |  |  |
| Negative Symptoms of psychosis |  |  | ns |  |  |  |  |  |  |
| Thin-ideal Internalization |  |  |  | **Y*** Intervention: postintervention *d* = 0.74, p < .05;  8 weeks, ns;  8 months *d* = 0.30, p < .05;  14 months *d* = 0.51, *p* < .05, compared to baseline.  Control: postintervention *d* = 0.36, p < .05;  8 weeks ns;  8 months ns;  14 months *d* = 0.41, p < .05, compared to baseline. | **Y*** Trial 1 EVERYbody (w/ expert - and peer-leaders): postintervention, *d* = 0.55, *p* < .05;  1-month *d* = 0.21, *p* < .05 compared to controls;  group x time interaction, *b* = −0.17, *t* = −4.72, *p* < .0001.  Trial 2 EVERYbody (w/ peer-leaders only): postintervention, *d* = 0.27, *p* < .05;  1-month *d* = 0.21, p < .05 compared to controls;  group x time interaction, *b* = −.24, *t* = −5.33, *p* < .001. | ns | **Y*** Intervention: postintervention *d* = 1.04 *p* < 0.001; 4-weeks *d* = 1.71 *p* < 0.001; 24 weeks *d* = 1.04 *p* < 0.001, compared to controls. |  |  |
| Body Dissatisfaction |  |  |  | **Y*** Intervention: postintervention *d* = 0.50, *p* < .05;  8 weeks *d* = 0.45, *p* < .05;  8 months *d* = 0.59, *p* < .05;  14 months *d* = 0.97, *p* < .05, compared to baseline.  Control: postintervention *d* = 030, *p* < .05;  8 weeks *d* = 0.48, *p* < .05;  8 months *d* = 0.36, *p* < .05;  14 months *d* = 0.83, *p* < .05, compared to baseline. | **Y*** Trial 1 EVERYbody (w/ expert - and peer-leaders): postintervention, *d* = 0.63, *p* < .05;  1-month *d* = 0.61, *p* < .05, compared to controls;  group x time interaction, *b* = −0.15, *t* = −4.10, *p* < .0001.  Trial 2 EVERYbody (w/ peer-leaders only): postintervention, *d* = 0.26, *p* < .05;  1-month *d* = 0.13, *p* < .05 compared to controls;  group x time interaction, *b* = −0.32, *t* = −5.72, *p* < .001. | **Y*^b^** Females Intervention:  Female Only group: postintervention, *d* = 0.67, *p* < 0.0001;  2-month ns;  6-month ns, compared to controls.  Females in Mixed Gender group:  postintervention, *d* = 0.49, *p* = 0.0015;  2-month ns;  6-month ns, compared to controls.  Males  Intervention:  Males in Mixed Gender group:  postintervention, *d* = 0.54, *p* = 0.0005;  2-month *d* = 0.33, *p* = 0.0308;  6-month *d* = 0.38, *p* =0.0134, compared to controls. |  |  |  |
| Bulimic Pathology |  |  |  | **Y*** Intervention: postintervention *d* = 0.54, *p* < .05; 8 weeks *d* = 0.39, *p* < .05; 8 months *d* = 0.55, *p* < .05; 14 months *d* = 0.85, *p* < .05, compared to baseline.  Control: postintervention ns; 8 weeks *d* = 0.44, *p* < .05; 8 months *d* = 0.43, *p* < .05; 14 months *d* = 0.68, *p* < .05, compared to baseline. |  |  |  |  |  |
| Eating Disorder Pathology |  |  |  | **Y*** Intervention: postintervention *d* = 0.64, *p* < .05;  8 weeks *d* = 0.49, *p* < .05;  8 months *d* = 0.35, *p* < .05;  14 months *d* = 0.78, *p* < .05, compared to baseline.  Control: postintervention *d* = 0.47, *p* < .05;  8 weeks *d* = 0.48, *p* < .05;  8 months *d* = 0.34, *p* < .05;  14 months *d* = 0.60, *p* < .05, compared to baseline. | **Y*** Trial 1 EVERYbody (w/ expert - and peer-leaders): postintervention, *d* = 0.56, *p* < .05;  1-month *d* = 0.42, *p* < .05, compared to controls;  group x time interaction, *b* = −0.24, *t* = −11.95, *p* < .0001.  Trial 2 EVERYbody (w/ peer-leaders only):  postintervention, *d* = 0.02, *p* < .05;  1-month *d* = 0.06, *p* < .05 compared to controls;  group x time interaction, *b* = −0.24, *t* = −11.95, *p* < .0001. | **Y*/ns FU2&6M** Females Intervention:  Female Only group:  postintervention, *d* = 0.46, *p* = 0.0029;  2-month ns;  6-month ns, compared to controls.  Females in Mixed Gender group:  postintervention, *d* = 0,51, *p* < 0.001;  2-month ns;  6-month ns, compared to control. |  |  |  |
| Male Body Attitudes |  |  |  |  |  | **Y*/ns FU6M**  Males  Intervention:  Males in Mixed Gender group: postintervention, *d* = 0.55, *p* = 0.0006; group;  2-month *d* = 0.37, *p* = 0.0167;  6-month ns, compared to control. |  |  |  |
| Body Shape |  |  |  |  |  |  | **Y*** Intervention: postintervention *d* = 0.76 *p* < 0.01; 4-weeks *d* = 1.27 *p* < 0.001; 24 weeks *d* = 1.06 *p* < 0.001, compared to controls. |  |  |
| Eating Attitudes |  |  |  |  |  |  | **Y*** Intervention: postintervention *d* = 0.79 *p* < 0.01; 4-weeks *d* = 1.37 *p* < 0.001; 24 weeks *d* = 1.19 *p* < 0.001, compared to controls. |  |  |
| Intuitive Eating |  |  |  |  |  |  | **Y*** Intervention: postintervention *d* = 0.80 *p* < 0.01; 4-weeks *d* = 1.25 *p* < 0.001; 24 weeks *d* = 0.82 *p* < 0.001, compared to controls. |  |  |
| Body Appreciation |  |  |  |  |  |  | **Y*** Intervention: postintervention *d* = 0.73 *p* < 0.01; 4-weeks *d* = 1.31 *p* < 0.001; 24 weeks *d* = 0.85 *p* < 0.001, compared to controls. |  |  |
| Meth-amphetamine Use |  |  |  |  |  |  |  | **Y*^d^** Intervention:  3 months *OR* 1.07, *p* < .05;  6 months, OR 1.11, *p* < .05;  9 months, *OR* 1.00, *p* < .05;  12 months *OR* 0.92, *p* < .05, compared to controls. |  |
| Condom Use |  |  |  |  |  |  |  | **Y* ^d^** Intervention:  3 months *OR* 0.95, *p* < .05;  6 months, *OR* 0.92, *p* < .05;  9 months, *OR* 1.28, *p* < .05;  12 months *OR* 1.04, *p* <.05, compared to controls |  |

*Note:* Y*(bolded) = significant result, ns = non-significant, FU = Follow-up, (number)W/M= number of weeks or months, Note: DASS-21 = Depression, Anxiety and Stress Scale, ATQ = Automatic Thoughts Questionnaire, CBT-Lit = Cognitive Behavioral Therapy Literacy Scale, OSSS = Online Social Support Scale, SSS = Stigma Stress Scale, QoL = KIDSCREEN-10 Index, ISMI = Internalized Stigma of Mental Illness Inventory, Short Form, SSMIS = Self-Stigma of Mental Illness Scale-Short Form, subscale apply/self-concurrence, ES = Empowerment Scale, SCOS = Stigma Coping Orientation Scales (Link et al., 1991), GHSQ = General Help Seeking Questionnaire, BHS = Beck Hopelessness Scale, Short Version, SISRS = Self-Identified Stage of Recovery Scale, CESD = Center for Epidemiologic Studies Depression Scale, PSP = Personal and Social Performance Scale, CDSS = Calgary Depression Scale for Schizophrenia, UCLA = UCLA Loneliness Scale (Version 3), MOS-SSS = Medical Outcomes Study Social Support Survey, SERS-SF = Self-Esteem Rating Scale - Short Form, MHCS – Mental Health Confidence Scale, SWLS – Satisfaction with Life Scale, AQoL-8D – Assessment of Quality of Life - 8D, PANSS = Positive and Negative Syndrome Scale, IBSS-R = Ideal Body Stereotype Scale – Revised, EDE-Q = Eating Disorder Examination- Questionnaire, PANASX = Positive and Negative Affect Schedule – Revised, PANAS = Positive and Negative Affect Schedule, PANAS-B = Positive and Negative Affect Schedule, SATAQ-4R = Sociocultural Attitudes Towards Appearance Questionnaire - 4, SDBPS = Satisfaction and Dissatisfaction with Body Parts Scale, BPSS-R = Body Parts Satisfaction Scale – Revised (Petrie, Tripp, & Harvey, 2002), MBAS = Male Body Attitudes Scale, IES-2 = Intuitive Eating Scale-2, UPE = IES-2 subscale Unconditional Permission to Eat, EPRER = IES-2 subscale Eating for Physical Rather than Emotional Reasons, RHSC = IES-2 subscale Reliance on Hunger and Satiety Cues, BFCC = IES-2 subscale Body-Food Choice Congruence, EAT-26 = Eating Attitudes Test-26, BSQ-8 = Body Shape Questionnaire-8, RSES = Rosenberg Self-Esteem Scale, BAS-2 = Body Appreciation Scale-2, DD = Disclosure Distress (≥4 for ‘In general, how distressed or worried are you in terms of secrecy or disclosure of your mental illness to others?’, rated from 1/not at all to 7/very much), AD = Attitudes to Disclosure (‘In general, how comfortable would you feel talking to a friend or family member [item 1; ‘... to a teacher or employer ...’ in item 2] about your mental health, for example, telling them you have a mental health diagnosis and how it affects you?’, from 1/not at all to 7/very much).

^a^ Kipela et al. (2016) found negative affect only decreased with the males in their mixed-gender group, not with the females in the mixed-gender group or with the female only group.

^b^ Kipela et al. (2016) found body dissatisfaction decreased after postintervention for both females and males, however, these effects were lost at two- and six-month follow-up for females.

^c^ Resende et al. (2021) found negative affect only decreased at the 24-week follow-up, not at post-intervention, or four weeks follow-up

^d^ These two results were reported in another study on the same intervention by Sherman et al. (2009).
